# Embracing a Sustainable Approach in Gynecology and Obstetrics: The Surgeon's Duty to Safeguard both Patient and Environment

**DOI:** 10.1055/s-0043-1772472

**Published:** 2023-09-08

**Authors:** Agnaldo Lopes da Silva Filho, Eduardo Batista Cândido, Mariana Seabra Leite Praça, Pedro Henrique Tannure Saraiva, Rívia Mara Lamaita, Michel Canis

**Affiliations:** 1 Department of Gynecology and Obstetrics, Universidade Federal de Minas Gerais, Belo Horizonte, MG, Brazil; 2 Department of Obstetrics and Gynecology, University Hospital Clermont-Ferrand, 63000 Clermont Ferrand, France

The detrimental effects of climate change on global health and wellbeing have become increasingly apparent and concerning. According to the World Health Organization (WHO), 1 climate change is projected to cause an additional 250,000 deaths per year between 2030 and 2050, due to malnutrition, malaria, diarrhea, and heat stress. Unfortunately, the healthcare sector also contributes to the problem, as it is a major source of greenhouse gas emissions, accounting for 4.6% of total emissions in 2017. 2 The operating theater is one of the most energy-intensive areas within healthcare facilities, with energy consumption rates three to six times higher than those of other hospital areas. 3 4 Medical waste production and the emission of harmful anesthetic agents during surgical procedures further exacerbate the environmental impact of healthcare. 5

To address these environmental issues, the concept of “green surgery” has emerged as a promising solution. Green surgery involves incorporating environmentally friendly materials and practices that conserve energy, reduce waste, and minimize greenhouse gas emissions, while still ensuring high-quality patient care. 4 5 In this editorial, we aim to provide a comprehensive examination of the importance of green surgery, with a specific focus on its application in gynecological and obstetric surgeries. We will explore the benefits, strategies, and challenges of incorporating green surgery into surgical practices, and provide insights into its potential to reduce the environmental impact of surgical procedures and promote sustainability in healthcare.

**Fig. 1:**
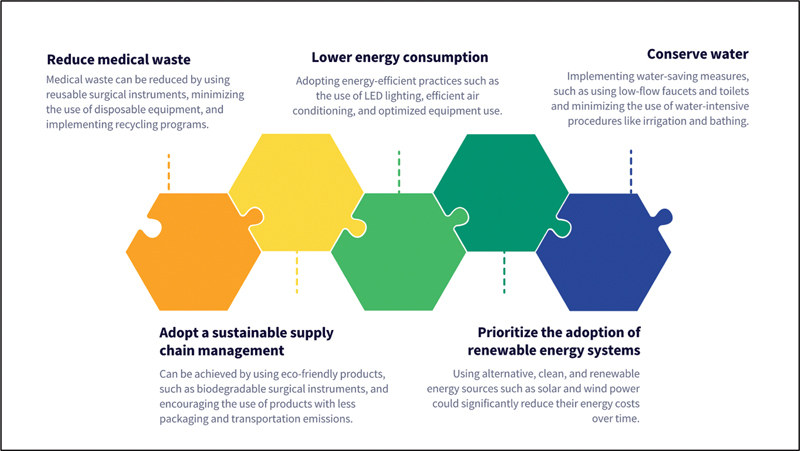
Greening Gynecology and Obstetrics: Key Strategies for Implementing Sustainable and Environmentally Friendly Practices.

Sustainability in health systems, as defined by the WHO, is a comprehensive approach that balances health outcomes with minimizing environmental harm. This approach recognizes the interconnectedness of health and the environment and stresses the importance of considering both when delivering healthcare services. 3 To achieve this goal, it is crucial to educate healthcare professionals about metrics related to greenhouse gas emissions, life cycle analysis, and strategies for reducing environmental impact in healthcare. The 5Rs rule—reduce, reuse, recycle, rethink, and research—can be effectively applied in medical practice, particularly in the operating room, to promote eco-friendly practices. 6 Adopting green surgery practices, which include minimizing waste, reducing energy and water consumption, implementing sustainable supply chain management, and prioritizing the use of renewable energy systems, is a crucial aspect of promoting sustainable healthcare ( Fig. 1 ). These efforts not only increase the sustainability of healthcare facilities but also align with the principles of environmental responsibility in the healthcare sector. By reducing their carbon footprint, medical facilities demonstrate their commitment to creating a more sustainable future for the planet. 5

The incorporation of green surgery presents a significant challenge due to the high costs associated with the use of green energy. Hospitals need to alter their processes and structures to achieve sustainability goals, which involves the entire chain of patient care. For instance, hospitals need to adopt new sterilization devices that emit fewer pollutants, consume less water, and deliver the same level of quality as disposable devices. The entire surgical team needs to be sensitized to the need to change the way it behaves in the use of resources, from proper handling of contaminated materials to energy savings. Additionally, surgical blocks should be equipped with modern LED devices to reduce energy consumption and automatic faucets to control water usage. 7 8 Unfortunately, such efforts may require a significant financial investment, particularly in underdeveloped countries. To overcome this challenge, it is essential for surgeons and anesthesiologists to lead the way in developing an ecological mindset. This involves acquiring greater knowledge of recyclable or reusable materials, rational consumption of greenhouse gases used in inhalational anesthesia, and energy-saving practices. 9 10 11

The Royal College of Surgeons of England has devised a comprehensive list of recommendations aimed at reducing the environmental impact of operating theaters. This list has been compiled based on an exhaustive review of all relevant guidance and published evidence and is available in the Compendium. 12

In the healthcare setting, sustainability efforts must go beyond the facility walls and begin from the preadmission stage. This involves the development of health promotion strategies and encouraging the use of eco-friendly transportation modes for patients, healthcare professionals, and medical supplies. Within the hospital, the implementation of renewable energy sources, optimization of energy systems, and reduction of disposable materials can significantly lower the carbon footprint and contribute to sustainability efforts. The use of intravenous and local anesthesia instead of anesthetic gases during surgical procedures is another environmentally friendly option. Implementing recycling programs and utilizing telemedicine can also play a crucial role in reducing waste production and supporting sustainability goals. 3 5 6

Hospitals in developed countries play a significant role in the generation of solid waste and greenhouse gas (GHG) emissions, accounting for 1% of the former and 2.1% of the latter on an annual basis. A considerable portion of these emissions is attributed to the operating room (OR) due to the usage of energy-intensive equipment and the emission of anesthetic gases. Additionally, the transportation and incineration of waste also contribute substantially to GHG emissions. It is estimated that the incineration of 1 kg of clinical waste generates 3 kg of carbon dioxide. Despite these facts, the WHO reports that a substantial amount of hospital and OR waste, ∼ 85% and 90%, respectively, is non-hazardous and similar to domestic waste, providing opportunities for recycling and reducing emissions. 6 Furthermore, gynecological surgeries can exacerbate this situation by producing a significant amount of waste, including disposable surgical instruments, gowns, drapes, and other single-use items, which can have a negative impact on the environment if not properly managed.

To mitigate this impact, green surgery focuses on reducing waste through the use of reusable surgical instruments and reducing single-use plastics. Reusable surgical linens and instruments are an eco-friendlier alternative as they result in lower waste generation and decreased expenses for landfill and incineration. A comprehensive evaluation of the production-to-sterilization process showed that the carbon footprint of reusables is more favorable compared with that of disposable options. For instance, the use of reusable surgical devices in laparoscopic procedures can save an estimated 122 kg of waste per case. 13 The use of reusable gowns can also significantly reduce waste output by up to 70%. 3

Minimally invasive surgery (MIS) is known for its benefits, but it also generates a significant amount of waste due to the reliance on disposable surgical instruments. To address this issue, healthcare providers need to adopt a comprehensive approach that includes minimizing material usage, transitioning to eco-friendly anesthetic gases, maximizing instrument reuse, and reducing energy consumption during off-hours in the operating room. The combined efforts of these measures can reduce the carbon footprint of an average laparoscopic hysterectomy by up to 80%. 14 A systematic review on the environmental sustainability of robotic and laparoscopic surgery has shown that the clinical benefits of robotic surgery may not justify its increased environmental impact. However, the review suggests several measures that could help reduce GHG emissions and waste in surgical practice. These include the use of alternative surgical approaches, reusable equipment, repackaging, surgeon preference cards, and staff awareness on open and unused equipment, as well as avoidance of desflurane. Implementing these changes could contribute to a more sustainable healthcare system. 15

In addition to considering changes in general medical procedures, it is crucial to incorporate sustainable practices in obstetric care as well. Maternity services should focus on developing and disseminating green and sustainable practices, which can be implemented quickly. 16 One area of particular concern is the OR and labor-delivery waste, which accounts for ∼ 70% of hospital waste. 17 Targeted solutions should be explored to address sustainability in this area. It is important for healthcare providers to prioritize environmental sustainability in obstetric care to minimize the impact of healthcare services on the environment.

Green surgery aims to minimize energy consumption through the implementation of energy-efficient technologies and practices, such as LED lighting and low-flow anesthesia machines, thereby reducing OR costs and enhancing the overall sustainability of healthcare facilities. 4 5 In addition, water conservation is a crucial aspect of green surgery, which can be achieved through the use of water-saving devices in the OR and minimizing water-intensive procedures. 3 Simple measures, such as avoiding repetitive water scrubbing and implementing automatic or pedal-controlled water taps, can effectively reduce water and energy consumption.

Sustainable supply chain management is a crucial aspect of green surgery, with the aim of minimizing the environmental impact of the production, transportation, and disposal of surgical supplies and equipment. A significant portion of carbon emissions produced by healthcare facilities arises from the procurement of drugs and medical devices, including the production methods, packaging, transportation to the hospital, and the energy and materials required for drug delivery. 6 In gynecologic and obstetric surgery, this objective can be achieved through the adoption of eco-friendly products, such as biodegradable surgical instruments, and the promotion of products with minimal packaging and transportation costs. 3

Optimizing efficiency, reducing waste generation, and conserving energy not only positively impacts the environment but also results in cost savings. A systematic review conducted by Sullivan et al. (2023) 5 evaluated the environmental and financial impact of quality-improvement initiatives in ORs. The review found that 90.9% of the 10 studies analyzed reported annual savings, ranging from $6,572 to $322,405 per year. Out of these, 45.5% reported savings per procedure, with cost reductions mainly attributed to reduced instrument processing or sterilization (54.5%). A few studies reported cost reductions from decreased supplies per case (27.3%) or less frequent use of individual instruments/supplies (27.3%). One study (9.1%) also included cost savings from labor, utilities, and instrument depreciation. In terms of patient outcomes, two studies (18.2%) reported positive results. One study found that changes in elective hand surgery procedures, including reducing disposables and modifying anesthetic approaches, resulted in a 96% satisfaction rate among patients. The implementation of a surgeon-specific scorecard with direct cost feedback and supply usage also led to reduced waste and had no negative effect on procedure times, length of stay, or complications.

Hospitals are significant consumers of energy, using more than 10% of the energy consumed for commercial purposes. 18 This high demand for energy presents an opportunity to explore alternative, clean, and renewable energy sources such as solar and wind power. By transitioning to these forms of energy, hospitals could significantly reduce their energy costs over time. Moreover, renewable energy systems are less vulnerable to disruptions than traditional fossil fuel systems in the event of accidents or natural disasters, making them a reliable and sustainable option for hospitals in Brazil. These technologies can help hospitals to operate efficiently and reduce their environmental impact while providing critical healthcare services to the population. 19

While there are numerous benefits to implementing green surgery, significant challenges must be addressed to effectively adopt this approach. These challenges include the lack of standardized guidelines and protocols, high costs associated with green technologies, and limited resources for their acquisition. Furthermore, insufficient knowledge among healthcare providers and inadequate training on how to utilize related technologies hinder the adoption of sustainable approaches in healthcare. Economic and financial challenges, including limited access to funding for implementing climate action, also pose significant barriers. Additionally, challenges related to policy, leadership, conflicting interests, and the lack of global awareness and advice on green surgery further impede progress. Finally, social and cultural challenges, such as the influence of adaptation and decision-making, as well as technological and infrastructure challenges and limited adaptive capacity due to a lack of human resources must also be addressed. 3

In conclusion, green surgery is an important concept in gynecology and obstetrics, with potential benefits in reducing the carbon footprint of surgical procedures, improving patient outcomes, and reducing healthcare costs. The implementation of green surgical practices, such as the use of reusable instruments, biodegradable materials, and energy-efficient electrosurgical generators, can have a significant impact in reducing the environmental impact of surgical procedures, as well as improving patient outcomes and reducing healthcare costs. However, there are still several barriers to the widespread implementation of green surgery, including the high cost of green technologies and the limited resources available for their purchase, as well as a lack of awareness and training among healthcare providers.
